# Plasma-Based Nanostructuring of Polymers: A Review

**DOI:** 10.3390/polym9090417

**Published:** 2017-09-05

**Authors:** Lan Thi Phan, Sun Mi Yoon, Myoung-Woon Moon

**Affiliations:** 1 Division of Nano & Information Technology, KIST School, Korea University of Science and Technology, Seoul 02792, Korea; 616003@kist.re.kr; 2 Life and Materials Science Research Division, Korea Institute of Science and Technology, Seoul 02792, Korea; smyoon@kist.re.kr

**Keywords:** plasma technology, nanostructuring, polymers, selective etching, wettability, battery, organic solar cells, biomaterials

## Abstract

There are various fabrication methods for synthesizing nanostructures, among which plasma-based technology is strongly competitive in terms of its flexibility and friendly uses, economy, and safety. This review systematically discusses plasma techniques and the detailed interactions of charged particles, radicals, and electrons with substrate materials of, in particular, polymers for their nanostructuring. Applications employing a plasma-based nanostructuring process are explored to show the advantages and benefits that plasma treatment brings to many topical and traditional issues, and are specifically related to wettability, healthcare, or energy researches. A short perspective is also presented on strategic plans for overcoming the limitations in dimension from surface to bulk, lifetime of surface functions, and selectivity for interactions.

## 1. Introduction

Following Faraday’s first scientific report of gold colloid in 1857 [1], researches in nanomaterial and nanotechnology have shown an abundant increase since 1990 [2], while the associated patents have shown a corresponding increase since 2000 [3]. In recent years, nanoscale discoveries in fundamental, mechanical, optical, electronic, magnetic, biological phenomena and properties have been reported from the aspects of chemical bonding, functionalized nanoparticles and quantum dots, or 2D materials, thanks to nanoscience. Nanostructured materials have assumed an undeniable role in scientific development, and enjoy strong participation in various industrial fields, such as the environment, medicine, biomaterials, energy, and materials.

Nanostructures can be classified as 0D-nanodot, 1D-nanowire, 2D-nanofilm (ex. Graphene), and 3D-nanoball or nanocoil [4] based on the dimensional size of nanoscale ~1–100 nm (1 nm = 10^−9^ m). Among the various nanostructure fabricating methods, the plasma-based method, since the first use by Irving Langmuir to describe an ionized gas as a fourth state of matter in 1927 [5], has presented many advantages as a research and industrial technique. Beside the uses for cleaning and sterilization [6,7] plasma takes effect on the surface or near surface, without any change in the bulk material properties, and results in many nanostructure morphologies. Various types of surfaces on different materials could be used for plasma processing, including plain, rough, and porous surfaces, or even the inner surfaces of tubes. The use of plasma firstly focuses on electronics, integrated circuits, microfluidics, or lab-on-a chip areas by precise plasma patterning methods, such as soft lithography [8] and laser based technique [9]. Recently, it is used in many fields where more nanostructures have been fabricated in various materials. Plasma etching for polymer is prominent in pattern formation in nanoscale by controlling the surface-to-volume ratio, surface energy, aspect ratio in geometry, light absorbance, surface functionalization, or size effects.

Plasma uses an extra energy source to energize atoms or molecules by high voltage, but the treatment process could be implemented under ambient temperature, implying it is facile for heat-sensitive materials. The results after treatment are highly reproducible, and are characterized by greater consistency than after chemical or mechanical processes. Furthermore, it is low-cost in operation, easy to use, and is considered an environmentally friendly process.

Two approaches for the plasma-based fabrication of nanostructures can be classified in plasma processing: bottom-up and top-down, mainly in a dry environment. The top-down approach is by treating the surface to obtain downscale, such as plasma etching, implant, or deposition; while the bottom-up process is by gaseous agents to create upscale objects, such as 2D layer, pore, nanowire [10] by PECVD, for example. In practical ways, the hybrid systems of top-down and bottom-up methods are also used simultaneously in some works, namely etching as a top-down method, and deposition as a bottom-up method would commonly be used in a single plasma process. In the scope of this work, plasma deposition and plasma growth are not focused on as a method for polymer nanostructuring.

So far, there are several review articles regarding aspects of plasma, from techniques [11,12] to classification [13]. Some reviews focused on plasma in combination with recent popular topics [14], such as bioapplication [15], medicine [16], biomaterials [17] wound care [18] and decontamination [19]. However, the full potential of plasma has yet to be revealed. In particular, after plasma was first introduced during research employing electrical discharge tubes from 1960, plasma-based nanostructuring on polymeric surfaces has emerged as a newly developing area with abundant applications [20]. The purpose of this review is to provide more comprehensive details, and different approaches for the issues that need to be understood in plasma for polymer nanostructuring and the role of this process applied in various research fields and related industries. In the following, Section 2 reviews plasma itself for nanostructuring, including a brief introduction of several recent plasma etching machine/methods related to the different methods of plasma generation. Moreover, several plasma parameters/quantities related to gas precursors, ion density, electron density, and more on etching parameters and numerical analysis are carefully introduced; Section 3 presents discussions on substrate material-plasma interactions. Plasma etching techniques are analyzed for the various nanostructures morphologies on different substrate materials with appropriate etching gas, plasma source/techniques. Several selectivity etching methods are reviewed in terms of impurities, crystallinity and inhibitor agents in the formation of nanostructures; Section 4 includes the strong contribution of plasma processing in many outstanding works. We review the surface functionalization by plasma in terms of changing the surface ratio, surface energy, aspect ratio, and anti-reflection properties for several application fields in the subsections; Section 5 concludes the review, and presents a short perspective on strategic plans to overcome the limitations in dimension from surface to bulk, lifetime of surface functions, and selectivity for interactions.

## 2. Plasma Technology for Nanostructuring

In this section, plasma for nanostructuring on polymeric materials is discussed in terms of formation, particles, precursor gas, and other physical parameters. Investigations are presented into plasma itself, the quantified factors, and analysis from simulation.

### 2.1. Plasma Generation

The scope of this work refers to low temperature and low pressure plasma, due to the low thermal resistance of polymers, and to prevent contamination and facilitate gas ionization. Plasma is created when gas molecules are provided with extra energy to generate free electrons from their outlet orbitals, or by collisions between gas molecules, particles, or free electrons [21]. Therefore, namely free electrons, charged particles, ions, and neutral radicals exist in bulk plasma. The plasma is generated by electrical energy in several ways. For DC plasma, high voltage is used to provide potential energy for background gas, then to release valence electrons to form discharge inside the chamber. In microwave plasma, the energy is generated from activating the positive and negative parts inside gas molecule at high frequency (~2.45 GHz) to generate free electron and ionize the gas. In RF plasma, included capacitively induced/inductively induced plasma, where the frequency is around 1–100 MHz, plasma generation is based on both potential energy and high frequency. In many systems, electron heating is used to trigger discharge avalanches or magnetic fields (magnetron system) is used to concentrate the ion and electron in a specific area.

### 2.2. Ion Etching

There are many species in plasma environment, including electrons, neutral particles, excited particles, radicals, ions (mostly negative), photons, or X-rays. These particles play an important role in the polymer etching process. Electrons would be the largest number among others in the plasma bulk due to the high kinetic energy and high velocity, and are the main agent for plasma self-generation. They usually distribute in the center area of plasma. However, ions with high momentum mostly concentrate on the sheath layer with thickness of several to tens of mm, locating right next to the cathode. The electrical field in this area might be 100 times higher than the center of plasma. Therefore, they function as the main agent for the etching process.

The ways in which ions interact with surface in bulk plasma or in ion beam make significant changes to the modified surface. In glow discharge, ions are generated from the bulk plasma area, accelerated in a very short sheath near the cathode, and then bombard, and etch substrate when it is placed onto the cathode surface. Ions from an ion beam (IB) system are accelerated onto a target area, and the etching effect occurs on these areas only. In general, IB etching by ion bombardment of inert gas, such as Helium (He), Xenon (Xe), or Argon (Ar), is considered to be physical etching, because no chemical interaction is observed on the target surface after processing. The process is ion milling, which is used widely as a common etching process, such as the FIB system, which is popular in scientific instruments for analytical techniques or thin film characterization methods. An improvement from sputter in plasma etching is RIE, which is conducted with chemically active ions and radicals to increase the etching depth, or enhance the sputtering rate. The main steps in the RIE process are: (1) formation of plasma and the reactive particle; (2) arrival of the reactive particle at the surface to be etched; (3) adsorption and chemical reaction of the reactive particle at the surface; and (4) removal of the volatile product molecule from the reactor [22].

### 2.3. Gas Precursor Behavior

For dry etching, precursor gas selections are very important. The electronegativity of gases changes the number of free electrons in the chamber, because high electronegative gas easily traps free electrons, and downsizes the number of charged particles. For polymer nanostructuring by plasma, reactive ion etching from O, F, S, Cl, or Br is the common selection (Table 1). Gases with low electronegativity but reactivity, such as O_2_ or N_2_, create high density of electron and positive ions, while F, Cl, or Br containing gases are much more electronegative, hence decrease the density of free electron, but increase the number of negative ions. The electronegativities are lower in the order of F > Cl > Br. The gas selection might be related to the density of ions (mostly positive ions), because it is the main agent for the etching process. The lower numbers of electrons, as the agents to sustain the plasma, cause lower stability, and hence require higher power to maintain this environment. The ion speed and trajectory are important factors for the etching process, and relate to nanostructure shape and etch rate, which will be deeply analyzed in the next section. In general, ions are usually strongly accelerated in the sheath area, and the trajectory is perpendicular to the Cathode surface. Therefore, they will approach the surface from the top, and bombard the surface vertically, rather than from other directions.

### 2.4. Etching Parameters

The selections of gas, substrate, power, and bias voltage cause changes in the resultant etching process, such as the etch rate, selectivity, anisotropy/isotropy profile, and uniformity. These parameters are fundamental factors for controlling the etching result. The etch rate shows the speed at which the surface is removed, which is usually in units of length or thickness to time. The selectivity shows the different etch rates of plasma responding to different substrate materials. In general, an etching mask (photoresist mask, or e-beam resist mask) is resistant to ions, neutrals, and radicals from plasma. They are traditional choices for plasma patterning, by adopting patterning technology that is popularly used in nano/microelectronics. The anisotropy/isotropy profile is also an attractive term, because it impacts the etching depth and size of nano structures. Isotropic etching usually is not expected for nano size etching, because the nano structure (pillar, needle-like pillar) is easily destroyed and collapsed. Therefore the etch depth is limited. The uniformity is a parameter that measures the thickness at certain points before and after the etching process, meaning the bias etching thickness, and could be derived through the etch rate. Those parameters within the real interactions between plasma and substrate are discussed further in the following discussions.

### 2.5. Numerical Analysis

Numerical calculation and simulation in plasma technology have been used for estimation of the physical parameters and chemical reactivity of particles in plasma chamber. Basically, all plasma calculations use the database of particle collision cross-section set of the gas [23,24] in ideal condition to calculate the energy, density, mobility, velocity, diffusion, and ionization coefficients. The Boltzmann and Monte Carlo methods are frequently used, but other modern theories, like mechanical fluid or R-Matrix are also broadly selected. In some theoretical assumptions, the etching process and other plasma processing have the same point, as they start with the building unit of ion on the surface [25], including other metallic elements or impurities. The redistribution and cluster formation follow to trigger the nucleation and develop new dots. For etching purpose, these dots function as an etching mask, hence forming a long structure on the surface by the shade effect. A work of high-density low-pressure inductively coupled plasmas simulation has been implemented to investigate the sensitivity properties of plasma toward the mixing ratio [26], considering 65 species in the CF_4_/CHF_3_/H_2_/Cl_2_/O_2_/HBr reaction set that was produced in electron impact reactions and heavy particle reactions. The work shows that increasing the CF_4_ ratio leads to more F atoms for etching, but lower Cl atoms density, therefore to a drop in the etching rate under certain conditions. The increasing CF_4_ ratio leads to the decrease of free electron density when mixed with Cl_2_, but sometimes enhances the number of free electrons (HBr). If H_2_ gas is added to the mixture, more positive hydrogen ions are present, and they have very low sputter yield, leading to a lower sputtering process. The increase of O_2_ ratio will always lower the etch rate due to greater oxidation of the wafer surface and lower plasma density. When HBr is added, the diluting effects of Ar or He will be triggered if mixed with F- or Cl-containing gases. This leads to less chemical etching, but more sputtering.

Roughening in nanoscale on polymer surface is the process observed widely in many reports using plasma technologies. The process is explained by the compositional changes in surface of the polymer, and a stiff thin layer is created, and strained under ion bombardments. A 3D modelling of roughness formation during isotropic plasma etching of 2 phases-consisting nanocomposite materials (polymer and graphite nanoparticles) and of a smoothing of homogeneous material was reported [27]. The report shows that the average roughness of nanocomposite is increased, while that of homogeneous material is reduced. However, the average height is decreased, in both cases by time, because of isotropic etching. Another work has reported more detail of how ions interact with the polymer surface by MD [28]. The simulation has been applied for 100 eV Ar^+^ plasma on PS surface. The work determined that a heavily cross-linked, dehydrogenated damaged layer was created on PS surface under ion Ar bombardment at the initial period, with high sputter yield of Carbon and Hydro atoms. However, the drastically lower sputter yield is achieved in steady state, and the roughness appeared due to the strains and mismatch between surface and bulk of substrate under ion bombardments.

Another work [29] simulated the plasma formation using the particle-in-cell, in which the kinetics of so-called “superparticles” includes many real particles of electrons, Cu neutrals, and Cu^+^ ions, moving in self consistent fields, which are calculated on the grid with Maxwell’s equation. The particle collisions are handled by Monte Carlo collision routines, and the code was developed using the resolution of one spatial but three velocity components. The experiments of DC arcing setup in a UHV chamber using Cu anode and cathode were presented. The setting parameters and results were used for simulation, and to compare with the simulation results. The simulation snapshots of time development of cratering by arcs show the crater formation by huge energetic plasma ion flux and nonballistic (thermal) energy deposition. The craters have complex shape in both cases of simulation and experiment, due to energetic ions accelerated in the plasma sheath potential.

## 3. Surface Nanostructuring by Plasma

The main focus in this section is plasma-based nanostructuring on materials. These are the processes of using plasma to create nanostructures, sometimes called nanotexturing or nanopatterning on target surfaces, which are mainly involved in the etching process. The techniques of nanostructuring by plasma etching have been used as a very effective way to create various types of nanopatterns on various materials, such as semiconductors, metals, and carbon-based or polymeric materials.

### 3.1. Plasma Nanostructuring Techniques

By considering how the plasma particles interact with each other and with substrate, one can control and achieve the desired patterns. The plasma techniques for nanostructuring can be divided into two groups: (1) based on the cooperation of setting factors in chamber or the plasma system, such as plasma source, gas, frequency, power, bias voltage, and distance between electrodes or magnet, to produce various ways to control the properties of plasma. The densities of ion, radical, electron, with their energy, momentum, or velocity, including moving direction, impurity, and electronegativity, would make a significant difference to the result of plasma processing. With the information of the selectivity, tilt angle of the substrate holder, the percentage of precursor or reactive gas, etchant gas, electrode distance, power, impurities, the nanostructure or even desired properties can be obtained or controlled; (2) Otherwise, other simple techniques can be used prior to, or to combine with plasma processing, for example mask constructed combinations of electron beam, photolithography, nanoimprint, nano self-assembly polymer, and laser-based techniques. The techniques are effective in controlling the shape, size, and uniformity of nanostructure array. The details in Table 1 offer a brief perspective of the nanostructuring process on various polymer substrates by plasma techniques.

### 3.2. Nanopatterning Techniques by Plasma Etching

**Figure 1:**
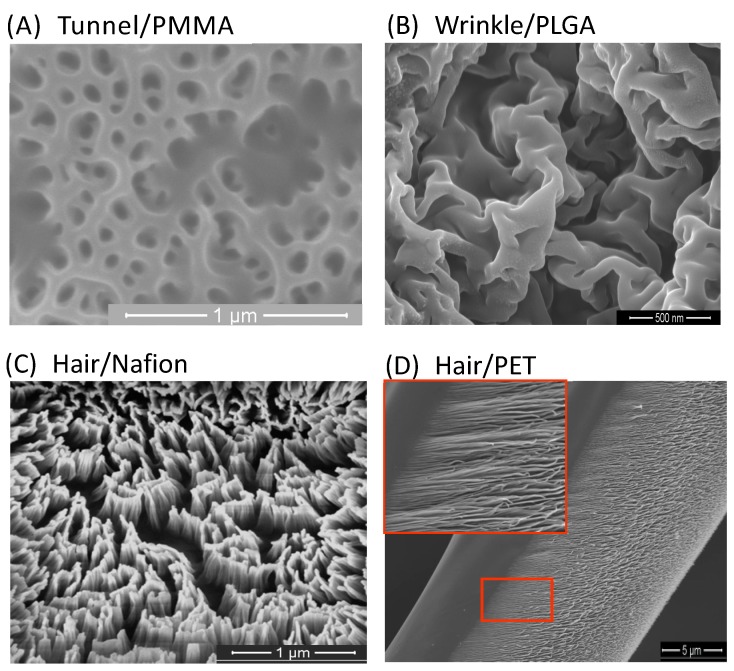
SEM images for nanostructured polymers by plasma ions (**A**) tunnel-like pores on PMMA surface; (**B**) surfaces wrinkles on PLGA; and (**C**,**D**) nanohairs on Nafion and PET with different ion beam directions to the surface: perpendicular and tilted angle, respectively.

Plasma etching is the most common method in the microelectronic industry, in combination with etching masks, such as photoresist materials, soft lithography, and laser-based technique [45,47,48]. The etching process increases the roughness of the surface, induces the nanostructure, and therefore increases the surface-to-volume ratio and changes the surface energy. This process provides the surfaces with new functions to apply widely in wettability application, biomedical, and energy related fields for electronic, supercapacitors, or batteries. Unlike uniform etching on the target surfaces in the semiconductor process, recent studies have been conducted to fabricate nanostructures ranging from dots, winkles, pores, or straight pillars to curly hairs in their configuration on materials [49]. Figure 1 shows images of several morphologies of polymer substrate after one step plasma nanostructuring. In general, the nanostructures observed in reactive plasma processing gas by surface evolution are usually nano-hole, nano-pore, nanotunnel, nano-cone, nano-pillar, or hair.

A work [50] on PMMA by FIB using ion Ga has successfully fabricated typical nanostructure morphology, such as hole, cone, and pore, by changing the acceleration voltage and ion flux (Figure 1A). The formation of these structures was explained by the interaction of ion Ga^+^ with PMMA not only on the surface, but also under the surface, because of the penetration of Ga^+^ into the bulk of PMMA. The interaction causes simple gaseous molecular species, which is associated with the degradation of the ester pendent group of PMMA. The gas concentration exceeds their solubility in PMMA, and become bubbles, which release from the bulk to the surface of PMMA. In combination with the process of bombardment on the surface, the process results in nano-holes, nano-pores, or tunnels.

The nanoscale roughness of winkles on polymer in plasma processing is considered for many applications, due to the high surface ratio in the resultant surface, as shown in Figure 1B. The high surface ratio is very attractive in many works, especially in the energy field, such as supercapacitors, and microelectronics. The stiff skin [51] formed in the initial period of ion beam processing on PDMS surface is simultaneously compressed under ion bombardments to give rise to highly nonlinear winkle patterns. Aiming to induce the wrinkle and control the strain and morphological evolution during ion beam deposition, acetylene (C_2_H_2_) IB has been deposited on PDMS in 30 s–30 min to form a stiff thin film of amorphous carbon [52]. The wrinkle formation is explained by the induced strain, and the mismatching strain in amorphous carbon film. However, even when ion beam was used, the chemical interaction and collisions between ion particles would occur, leading to ion scattering, and inducing the bombardment and strain in different directions.

Hairy nanostructure is frequently obtained in plasma treatment with anisotropic etching and high selectivity (Figure 1C,D). Even though the above morphologies are created in perpendicular ion beam, one actually could control the ion trajectory for slanted nanostructures. Faraday cage placing onto the cathode to lift up the sheath area is one way to control the ion trajectory. Because the electrical field is always perpendicular to the top mesh of the cage, which means that the ion trajectory moves perpendicularly to the mesh, it is possible to change the angle of ion—the surface interaction—by controlling the slanted surface angle or mesh angle.

### 3.3. Surface Energy Changes by Plasma Processing

Under the physical and chemical interactions with gas plasma, the surface boundary will change the chemical structure and shape. With the changes in chemical structure, the intermolecular bond in interaction with other materials will be modified. Usually, the surface energy is based on polar or non-polar groups, which anchor on the surface boundary. The polar group will cause stronger surface energy and hydrophilicity. For example, polymer under oxygen plasma usually becomes hydrophilic, because there are several oxygen-containing polar groups created in the etching process. A plasma etching of PET in 60 min [53] has changed the surface hydrophobic to superhydrophilic with the formation of nanopillars, and many polar groups, like –OH, –COO–, and –COOH. However, the thermal annealing would be used to robustly reduce the surface energy, and recover the original group on the surface. The surface after annealing would become superhydrophobic with CA ~ 174°. However, halogen-containing plasma etching usually induces the halogen group, which is less wettable than the carbon hydrogen group (non-polar group) [54]. The surface energy modification will create new function to substrate, and can be applied in many works relating to wettability, biocompatibility, or adhesion. For example, the polar group formed on polyvinyl acrylate (PVA) in atmospheric plasma has increased the surface energy from 37.1 to 60 mJ·m^−2^ [55].

The nanostructuring process with the modification of surface energy is one of the functionalizations for materials. Besides that, the increase of surface area, aspect ratio, directional nanostructure, anti-reflection, and size effects of nanostructure also bring materials many functions that are analyzed in the next sections.

### 3.4. Selective Etching by Inhibitor, Impurities, and Crystallinity

**Figure 2:**
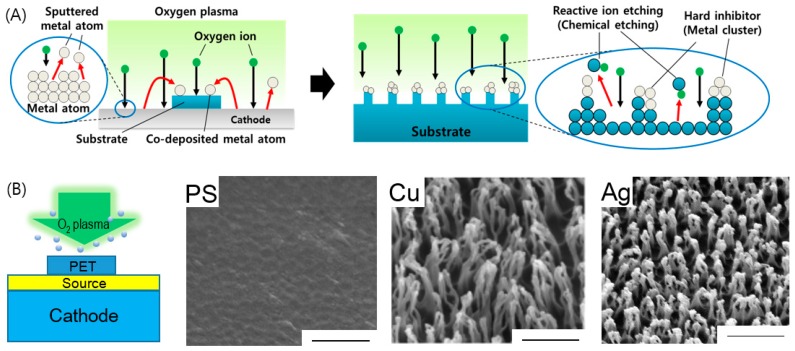
(**A**) A schematic for pattern formation during the preferential etching on the polymer under oxygen plasma treatment; (**B**) A schematic of the experiment material co-deposition on the PET covered on the cathode in 30 min with different sources, included PS designed to show induce, Ag, Cu. The scale bars represent 500 nm. Reprinted with permission from [32]. Copyright 2014 Wiley.

The hairy nanostructure formation was explained by the co-sputtered metallic self-mask covering the polymer surface; for example, the metal particles deposited on the surface from the early stage of the etching process (Figure 2A) [32]. As more metal element is co-deposited along with oxygen plasma irradiation, discrete metal islands grow, and coalesce into larger metal islands, which later act as an etching mask on polymeric or semiconductor surfaces. Once the size and distribution of the etching masks are fixed, the size and density of the nanostructures are also determined. Then the simultaneous deposition of a third species creates regions on the surface that are not etched, while the regions with no third species are etched, causing etching anisotropy, and resulting in columnar nanostructures. Consequently, nanostructures with the high aspect ratio (the ratio of the height over the width) could be fabricated on carbon-based or silicon-based materials. Note that nanoscale metallic clusters decorate the top of the polymeric nanostructures, forming a hetero-nanostructure. Several factors have been considered for nanostructuring on polymeric surfaces, as shown in Figure 2B. It was found that no nanostructure appeared on the PET substrates in the case of stainless steel cathode covered by PS, which has only hydrocarbon molecules, when they have been etched by oxygen plasma as usual, due to the absence of supply of co-deposited metallic components.

Other experiments [56] confirm the results, and determine the relationship between metal concentration and nanostructure intensity. A PET substrate attached with a stainless steel strip on the side has been processed by oxygen plasma. The nanopatterns that have been observed on the PET substrate are dependent on the distance between their locations on the PET substrate, and the stainless steel location. The experiment shows that metal concentration is a critical factor in nanostructuring. The greater the metal concentration (near stainless steel) available, the higher the nanostructure intensity that appears. The direct proportion of metal concentration to the nanostructure intensity has proved the role of metal concentration in the nanopatterning process.

Since the selective etching mechanism needs a metal mask for blocking the chemical or physical etching reaction of oxygen plasma ion with polymeric materials, the ion induced yielding of metallic mask materials, as etching resistance materials, should be a very important parameter. Experiments on the etching resistance effect have been conducted for Ag, Cu, Pt and Si, which have different etching rates or yields under the same plasma environment of oxygen in 30 min. The final nanostructures differ in concentration intensity, height, diameter, and shape. Similar result nanostructures have been observed when the substrate materials laid on stainless steel cathode in oxygen plasma were changed. Substrate of different crystallinity, such as amorphous polymeric (PMMA and PET), good crystal—carbon fiber, and perfect crystal—diamond, have been used for etching in oxygen plasma [56]. It was clearly shown that nanopillars have been fabricated on polymeric amorphous substrate in a very short time (3 min), rather than nanodots on carbon fiber and diamond substrate, and they transited into pillar or hairy structures in 30–60 min. The substrates were chosen for the carbon material in different crystallinity, indicating the different bonding energy between molecule and atom in different crystal. The strong bond in well-defined crystal causes high selectivity of plasma particles, due to plasma preferentially interacting with weaker crystal, such as polymer or amorphous. The other point to explain the different etching rates on those substrates is based on the effective carbon content in materials. In a report on the effects of polycrystallinity in nanopatterning by ion-beam sputtering, Yoon et al. [57] found the dependence of the growth of ripples on grain structure (size) in highly anisotropic fashion. HOPG and NG were used to show the difference in polycrystallinity. The pattern formation was ripple shaped, but the mean uninterrupted ripple length of HOPG was smaller than that of NG, due to the smaller grain size of HOPG than NG. Other results have been reported to facilitate various nanopatterns by applying variable sputtered geometry: dual ion beam sputtering, and sequential ion beam sputtering. The nanopattern formation by IB sputtering is affected not only by these conditions, but also by impurities. Macko et al. [58] observed that metal impurities resulting from the sputtering process induce patterns. The nanodot/hole and ripples on Si (100) were fabricated with the intentional co-sputter deposition of stainless steel, while no patterns were formed without co-sputtered steel under the same sputtering condition.

## 4. Applications

A huge effort has been made to exploit the large applicability of this fourth matter in science and technology. The plasma sterilizing machine has been popularly used for healthcare by antimicrobial radicals introduced from air or oxygen plasma. Plasma cleaning of surfaces is usually employed in removing grease, oil, oxides, or silicone by vaporizing the contamination, and in surface gluing or printing before the main treatment. Ion bombardment can be used in the process for cleaning/cutting, or even nanostructuring on target surfaces, with tuning the power of radicals. After the removal of surface components, the surface is activated with high surface energy, and the surface chemical characteristics are changed for strong gluing and high hydrophilicity, or sterilization of the target surface. With plasma polymerization of these materials, a thin layer is coated on the surface, with new functions of hydrophobicity/hydrophilicity, protective barrier against wear or friction, or gas barrier. The process has been applied to fine mechanical transmissions, medical devices, headlights/reflectors, textiles, and water/oil separator in biochip manufacturing, and sensor manufacturing. In this review, various applications are explored with plasma treatment for nanostructuring, in term of the functionalization of surface energy, roughness, aspect ratio, surface ratio, size effects, and light absorption in three subsections, as follows.

### 4.1. Wettability

Nature is the mother of ideas when people use it to serve their daily lives. Mimicry of nature has revealed many interesting functions for nanostructured materials [59]. Some of natural insects or plants can manipulate the micro/nano structure and surface energy on the body surfaces to obtain an extremely amazing efficiency in water collecting or self-cleaning. Examples include the self-cleaning and hydrophobicity of the lotus leaf [60], the dewing ability of Namib’s desert beetle for water harvesting in the African desert [61,62], and the adhesion between a lizard’s feet and a wall [63]. Mimicking nature, people have created many things useful for living, such as hydrophobic fabric [64], anti-fogging glasses, anti-icing glass used in cars [65], and dehumidifiers [66]. Some typical solutions for recent problems suggested from wettability have been proposed, included oil-water separation or self-cleaning, anti-fouling, anti-fogging and anti-icing surfaces [67].

To date, a vast number of researches on the wettability of plasma treated surfaces have been discussed and reported [68,69,70,71], in efforts to control the surface energy and nanoroughness. The substrates used for mimicry have been metal, semiconductor, or polymer, but the majority of reports belong to soft substrate. The trends have focused on multi-scale interfacial and surface energy cooperation, showing some popular environmental topics, such as oil leakage/spill, and various interesting approaches to domestic products, such as textile (hydrophobic coats), anti-fogging, anti-icing glass/porcelain, and frost-resistor. Recent trends are the arrangement and orientation of micro- and nano-structures that may control wetting states and liquid motion tendencies.

#### 4.1.1. Nanostructured Surface with Special Wettability

Control of the surface energy of materials is crucial the water/oil separation, showing water and oil restriction or absorption of materials, respectively, and vice versa. As discussed in Section 3, the surface energy and surface roughness would co-operate to control the liquid droplet contact angle [72], such as the interaction between materials and the oil or water in air or water. They should have a low surface energy close to that of oil, or have a high surface energy close to that of water, but be in solid state, and be durable enough against strong waves and wind, and large amounts of oil in the ocean. This process could be conducted with some specific surface structures (rough or nanostructured surfaces), and cooperate with low surface energy material deposition. Both of these could be made effectively in plasma environment.

It is well known that when a water droplet is placed on a solid surface, the surface energies of solid/vapor ($\gamma_{S}$), liquid/vapor ($\gamma_{L}$), and liquid/solid ($\gamma_{I}$) are in equilibrium state. Under stationary equilibrium, Young’s law has been applied for an equilibrium contacting angle (CA), $\theta_{e}$, on a flat surface, as, $\cos\theta_{e}=\frac{\left(\gamma_{S}-\gamma_{I}\right)}{\gamma_{L}}$. As the solid surface has roughness either in nanoscale or microscale, Young’s law can be modified by considering two proposals suggested by Wenzel and Cassie-Baxter. In a Wenzel state with the surface roughness factor, *R*, the equilibrium CA can be expressed as, $\cos\theta_{app}^{w}=R\cos\theta_{e}$. In a Cassie-Baxter state with a solid fraction of ϕ (= partial wetting area divided by projected area), CA is defined as $\cos\theta_{app}^{C}=\phi \left(\cos\theta_{e}+1\right)-1$. In both cases, the apparent CA considering its geometrical roughness strongly changes with the dependency of the surface roughness at nano-, micro-, or complex scales. With difference in surface morphology and surface energy, there are several approaches to oil and water separation: [73]

- (Super)hydrophobic–(super)oleophilic materials
- (Super)hydrophilic and underwater (super)oleophobic materials
- (Super)hydrophilic- and in air (super)oleophobic materials
- Smart materials with switchable wettability
- Separation oil/water emulsion

(Super)hydrophobic–(super)oleophilic materials provide a surface that lets oil penetrate easily through, while repelling or restricting water penetration, thus separating oil and water mixture. In this case, the surface has a low surface energy by decoration with a chemical compound [74]. The steps in this approach consist of (1) surface morphology modification, aiming to create roughness or nanostructure; and (2) surface energy modification, aiming to deposit a thin film of low surface energy chemical, popular on a rough surface.

The (super)hydrophilic and underwater (super)oleophobic materials are applied to allow water to penetrate the surface, but block oil from penetrating. The mechanism of this case is based on the hydrophilic properties of substrate, and therefore creates a thin water film to protect substrate from oil. Notice that oleophobicity will not occur in air when water film on the surface is not available. The third type of (super)hydrophilic and in air (super)oleophobic materials have a very different mechanism for oil/water separation. Some surfaces can simultaneously show hydrophilic and oleophobic properties based on a favorable interaction with polar liquid, and tend to repulse non-polar liquid [75]. These are usually made by flour surfactant exposed on the top of the surface. Smart material with switchable wettability is an interesting field, where the wettability of material could be changed in a large range from hydrophilicity to hydrophobicity, and oleophobicity to oleophilicity, respectively. The last one is water/oil emulsion, which is still a big challenge, because most water/oil separators cannot operate at too small a size of oil drops of around tens of micrometer. However, the fact is that when spilt, most crude oil blends will quickly emulsify with water, creating a stable mousse that presents a more persistent cleanup and removal challenge.

**Figure 3:**
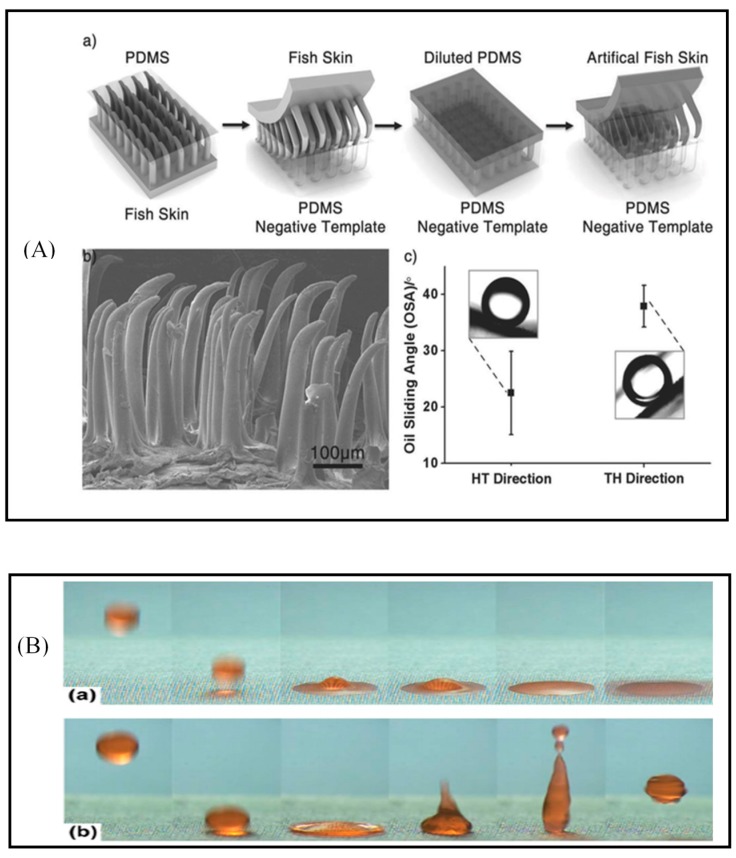
(**A**) (a) Illustration of the fabrication process for artificial fish skin; (b) SEM image of artificial fish skin fabricated by toluene-diluted PDMS; (c) Chart of anisotropic oil sliding angle (OSA) on oxygen-plasma-treated PDMS fish skin. The OSA values are 22.5° ± 7.3° along the head tail (HT) direction and 38.7° ± 3.7° along the TH direction. Reprinted with permission from [76]. Copyright Wiley 2013; (**B**) Bouncing behavior of water droplets on the upper image: (a) pristine and (b) nanostructured surfaces: superhydrophobic silk fabric. Reprinted with permission from [80]. Copyright RSC 2014.

The potential substrate falls into various types, from stiff to soft or porous materials, such as metallic, fabric, polymer, or foam-based substrates. The metallic substrate has the advantage of good mechanical properties, and could be durable in hazardous environments. However, the interaction with plasma of metallic substrate would be weaker than that of soft substrate. Other types of fabric or polymer would be more suitable and effective objects for nanostructured or rough surfaces, after surface energy modification. Cai et al. [76] were inspired by the unique oleophobicity under water of the skin of the Filefish *Navodon septentrionali*. They mimicked the oriented hook-like microstructure on fish skin on a PDMS layer, and used O_2_ plasma treatment to give the PDMS hydrophilic but anisotropic underwater oleophobicity (Figure 3A). The hydrophilic PDMS explains the underwater oleophobicity, but the pinning of oil in the direction from tail to head was explained by the hook-like microstructure. The principle is demonstrated on commercial cloth. This work could guide the mass manufacture of inexpensive high surface-energy materials for oil transportation, oil collection, and oil repellant coating on ships or oil pipelines.

#### 4.1.2. Self-Cleaning, Anti-Fouling, Anti-Fogging and Anti-Icing Surface

Amphiphobicity, such as the superhydrophobic and superolephobic properties of the surface, is becoming more popular in many applications, because the repellence of water or low surface energy liquid from rough substrate surface keeps the surface dry, which maintains a clean surface, and expands the lifetime of machines and materials. The low surface energy leads to low contact area between liquid and material, and therefore makes the materials self-cleaning, anti-fouling, or even anti-icing, but not anti-fogging, which needs high surface energy. This prevents severe detriment caused by the fouling of oil or water. The properties are originated from nature for the same purposes. The water/oil repellence ability of lotus leaves suggests special raincoats or fabrics that are always clean, or easy to clean from foul water or oil. Fish scales suggest the oleophobic but superhydrophilic abilities for anti-bio fouling surfaces. Many oleophobic surfaces in water or without water have been fabricated by plasma [77] Some have good durability [78], or scratch-resistance [79]. A self-cleaning or anti-fouling process is also useful in drinking-water reservoirs and distribution systems, and other machinery devices (membrane and surface) relate to preventing contamination, and destruction from biofouling agents. Oh et al. [80] have reported a high-aspect ratio nanostructured silk fabric with dual contract wettability using O_2_ plasma (Figure 3B). Superhydrophobicity was obtained on the plasma-treated surface, while the opposite side, or body-contacting side, retained pristine superhydrophilicity in the silk fabric. The dual wettability of silk fabric would be very useful and comfortable for people if available in the market. The results show that up to a point, the silk fabric after treatment could endure a heavier weight, but smaller extension.

Mundo et al. [81] also used a capacitively coupling plasma reactor to fabricate high rough nanotextured PS surfaces ranging from the good adhesive to the good slippery. The work could be applied with other properties of PS, such as transparency, or elasticity as plastic for exterior uses. Another useful application [82] is self-cleaning lyocell fabric, with good moisture absorbency. This can be used as shirt fabric that is convenient in hot weather and on rainy days, with the possibility of self-cleaning and antifouling. The polymer substrates also use plasma nanotexturing to control the superamphiphobic to amphiphilic properties with ordered hierarchical roughness [83]. In addition, anti-icing properties could be achieved by adopting superhydrophobicity to obtain non-condensing surfaces [84]. This is meaningful in transportation, especially for airplanes in low temperature. In cold air, the highly rough surface makes obstacles for ice crystallization, restricting the condensation of snow or frost on the top of nanostructure, because of the very low contact area. Therefore, the drops of ice or frost are unable to stick on the surface over time when their size increases gradually, and gravity force removes them away, leading to anti-icing or ice removal. Mobarakeh et al. [85] have deposited a superhydrophobic thin film on aluminium oxide substrate using RF-PECVD with HMDSO as precursor gas. The thin film shows an ice adhesion strength of hydrophobic coating that is 3.5 times lower than that on pristine Al substrate. After 15 times of icing/de-icing cycles, the thin film kept its icephobicity, and the ice adhesion strength was even 1.4 times lower than that of pristine Al.

In contrast to hydrophobic application, anti-fogging was obtained from a (super)hydrophilic surface, where the surface energy and the nanostructure/roughness were well developed. Yu et al. [86] have treated soda-lime glass by using a sacrificial SiO_2_ layer on glass substrate before CF_4_ plasma treatment to form nanostructure on glass substrate. The surface just having a nanopillar-like structure becomes superhydrophilic for anti-fogging. The surface could shift to superhydrophobic by an HMDSO deposition with 10 nm thickness. The surface with both superhydrophobic and superhydrophilic properties has been used in dewing for dehumidifiers. The same technique is also found on the Namib’s desert beetle, when people tried to discover how this insect could survive in the desert. Anna et al. [87] used a patterned sample using (super)hydrophobic/hydrophilic properties to investigate the process of harvesting water from humid air via dewing, showing the contrast to a fog basking method, where the most efficient surface consists of hydrophilic islands surround by hydrophobic background, rather than hydrophilic surfaces.

Yu et al. [88] have reported a Si surface with ultra-low reflective nanoscale hierarchical structure fabricated nanograss-like structures, by adopting CF_4_ plasma etching, and the hydrolysis effect of Al coating. This surface not only has the potential to capture photons to increase the efficiency of solar energy devices, but also the roughness to improve self-cleaning properties. Mundo et al. [89] also reported a method to fabricate a long-lasting anti-fog PC surface by plasma treatment. The PC substrate has been etched by O_2_ gas plasma to ignite nanotextured etching on the PC surface, and deposition by a low surface energy layer of HMDSO/O_2_/Ar mixture. The gas flow rates of HMDSO and Ar have been kept the same, while the gas flow rate of O_2_ has been controlled to change the wettability of surface. At high gas flow rate and high plasma power, the surface becomes superhydrophilic, therefore tailoring a permanent anti-fog modification on a commercial transparent PC. However, the durability of superhydrophilicity is particularly rare in related literature. Once plasma treatment achieves high yield, commercialization would be variable for transparent materials with special wettability, because of the wide demand and various applications. For example, dual superhydrophobic and anti-fogging surfaces also offer benefit to short-sighted people who have to wear glasses every day, or to people using goggles for special activities, such as winter sport, military action, or underwater activities.

### 4.2. Bioapplications

Recent trends in the bio-application are more related to new and modern technologies applied for healthcare, such as biomedical, bio-sensing for small biomolecule (DNA, protein, glucose, etc.), tissue engineering, artificial implants (3D imprinting), and cell culture. Biomedical uses have been applied for a long time to cure or remedy diseases. This usually goes with some prerequisites, such as biocompatibility, meaning a non-viable material is used to interact with biological systems, and result in the clinical success of medical devices. The biomaterials have been made of hard or soft materials, such as metal or polymer, depending on the organism that they support, such as bone, skin or soft organ; but they need to meet the standard of physical or mechanical properties appropriate to the intended surface or bulk functions, namely wettability, permeability, biodegradability, strength, flexibility, and fatigue resistance.

Most techniques using plasma in biology focus on etching (nanostructuring), deposition, sterilization, functionalization, and biofilm formation. Biomedical materials, for these purposes, are the combination of plasma etching and deposition in gaseous plasma ion implantation, metal ion implantation, dual metal and gas plasma ion implantation, and PIII&D [90]. Proper plasma treatment will bring material a suitable function and properties. Wettability is the most fundamental property among various intended functions that the surface is assumed to be charged for. By controlling the surface energy and nanostructuring morphology, many bioapplications have been implemented. Here we discuss some works on the hydrophobicity and nanostructure for minimalizing the platelet deposition for blood-compatibility or non-thrombogennicity; the wettability and aspect ratio of nanostructure could be used for isolation of cell to environment, or the hydrophobicity for biomolecule immobilization.

**Figure 4:**
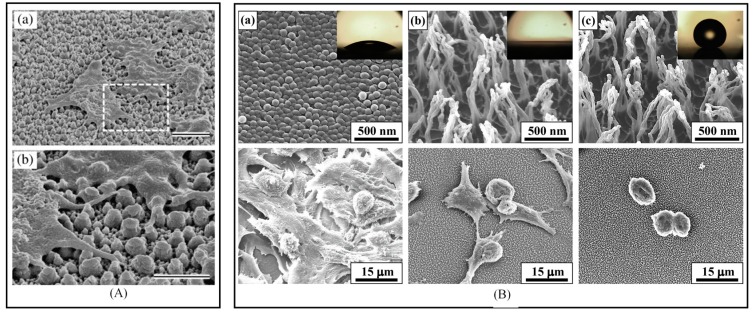
Plasma processing for bioapplications. (**A**) SEM image of adherent platelets on “F-DLC on dual.” (a) The outer shape of adherent platelet on dual rough surface and (b) detail of contact between platelet pseudopodia and micron-nano posts. Bar indicates (a) 1 μm and (b) 2 μm. Reprinted with permission from [92]. Copyright Elsevier 2013; (**B**) SEM images of (top) plasma-treated surfaces and (bottom) the adhesion behavior of mouse liver cancer cells on (a) a hydrophilic surface after 1 min of oxygen plasma etching; (b) a hydrophilic surface after 30 min of oxygen plasma etching; and (c) a hydrophobic surface after 30 min of oxygen plasma etching. The insets show the corresponding optical images of water droplets on each surface. Reprinted with permission from [99]. Copyright RSC 2013.

Blood compatibility is the first requisite issue for biomaterials [91] for minimal platelet deposition. Hasebe et al. [92] have fabricated a film for blood-contacting medical devices, using plasma of a C_2_H_2_ and C_2_F_6_ mixture gas for hydrophobicity and non-thrombogenicity of a nanoscale dual rough surface. The gas flow rate, plasma power, and coating time using this mixture will be optimized to obtain 50 nm thickness with a high hydrophobicity for F-DLC coating. Platelet adhesion and activation of this coating was checked with DLC coating (Figure 4A). The similarity in the adherent platelet and platelet-covered area per unit area in both F-DLC and DLC is the clue that shows the non-thrombogenicity of F-DLC based on the widespread clinical use of DLC coating for medical devices. The study opens the potential application of this material to clinical use, such as temporary blood-contacting devices. They also concluded the improvement of DLC and Titania coating layer, by using a block copolymer deposition with low surface roughness.

Plasma treatment used in tissue or cell culture engineering is another interest in bioapplication [93,94,95,96,97]. Intranuovo et al. [98] have investigated the behavior of osteoblast-like cell on a micro/nanopatterned coating that was deposited by RF plasma in a CVD system. The work was successful in creating micro/nanopattern on PET substrate by C_3_F_6_O and C_2_F_4_ gas plasma with different roughness. The results proved that topography is a decisive factor in mediating the cell-material interactions. The interesting aspects are the higher osteoblast-like cell adhesion, and spreading on the taller micro/nanorough coatings. On the other hand, it was observed that the cell usually prefers to adhere on smooth surface, rather than rough surface (Figure 4B) where they cannot move easily, and usually compact themselves, instead of stretching on the surface, resulting in the isolation of cell from environment [99].

Protein adsorption on surface has been considered in the relationship between nanoparticle size, nanoparticle density on surface in cooperation with plasma polyoxazoline polymerization [100]. Another method for direct covalent biomolecule (protein) immobilization has been investigated by Tsougeni et al. [101] on nanostructured PMMA substrate. A number of functional groups attached on a needle bed-like nanostructured surface were achieved by using O_2_ plasma treatment and an annealing step for long lasting hydrophobicity and stable chemical functionality. The substrates were checked with experiences of the immobilization of biomolecules RgGs and Immunoassays. This showed a chemical functional stable for a long period of 1 year in ambient storage, and would be used for the development of immunochemical assays for the sensitive detection of C-reactive protein and salmonella lipopolysacchatides.

### 4.3. Energy Applications

The energy field is one issue that has attracted a great deal of interest, with a long history of research. Human living depends predominantly on energy-based goods for electricity or oil. Even though large achievements have been obtained, there are several issues that should be addressed, such as environmental pollution, and the convenience and durability of energy sources. Furthermore, portable energy storages are a fast developing trend to cover the gap between indoor and outdoor energy. Lithium battery, solar panel, fuel cell, and photovoltaic research areas are becoming crowded. Capacitors and supercapacitors are also highly attractive trends, because of their quick and powerful recharge/discharge abilities in real time.

Plasma-based technology has provided facile methods for processing parts/components (electrodes, [102] and anodes [103,104]) of portable energy storage. The treatment brings new electrical, mechanical, and thermal properties, which are very effective in improving the performance of the materials. The increases in quality parameters of these devices using plasma obtain significant results, such as efficiency, energy density, lifetime, and reusability (rechargeability). Some discussions based on the control of nanostructure with the aspect ratio, the surface area, hydrophobicity, or even the selectivity of deposition are presented below.

**Figure 5:**
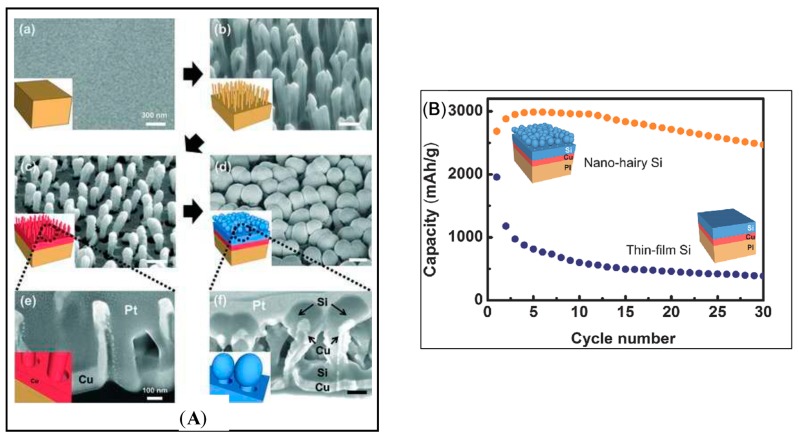
Plasma in energy applications. (**A**) Direct integration of nano-hairy Si anode on a nanorod array polymer substrate: (a) pristine PI substrate before CF_4_ etching; (b) well-arrayed PI nano-hairy structures after CF_4_ PECVD etching on pristine PI; (c) Cu (200 nm) as the current collector deposited by thermal evaporation; and (d) Si deposited (300 nm) as the active material by thermal evaporation on the sample (c); (e) A cross section image of sample (c); and (f) a cross section image of sample (d), both prepared by FIB cutting after Pt coating; (**B**) Capacity on delithiation step of Si on pristine and nano-hairy PI tested using a half cell. Reprinted with permission from [109]. Copyright 2014 Wiley.

Batteries are a fast expanding field in energy application, in particular secondary (rechargeable) batteries, because of the growing demand for the outdoor usage of portable devices. Among primary and secondary cells (or non-rechargeable and rechargeable batteries), a number of advantages of the portable battery could be listed to explain the attraction of these devices, such as long lifetime, lighter weight, safety, high energy density, and low self-discharge energy. In recent years, Lithium based batteries have been the main topic of many researches [105,106,107]. The demand for bendable, light weight, and cost-effective polymer-based electronics is increasing. Recently, extraordinary cycle stability for capacitors on polymer substrate has been reported [108], and it opens a wide pathway to batteries. Jung et al. [109] have reported the fabrication of a Lithium-ion battery with nano-hairy electrode with several layers on polymer substrate. Plasma CF_4_ gas has been used to etch PI substrate to form a nano-hairy layer to increase the surface area of the electrode. Thermal deposition of Cu on the flat bottom and along the hairs has been previously prepared to create current collector. After that, thermal deposition of Si was implemented to create an active layer for the integration scheme. The lithiation process is the last step for finalizing anode for the li-ion battery (Figure 5A); the process facilitates the kinetic lithiation process, and the strain accommodation process. The capacity of the nano-hairy Si anode has been significantly increased, along with the cycle life and rate capabilities, compared with a conventional Si thin film anode on pristine PI, by preventing mechanical failure (Figure 5B).

Fuel cells absolutely attract environmental protection energy resources, because of their high degree of clean, green energy, high efficiency and available fuel [110]. This energy offers potential in applications for home, transportation, or even rockets. There are many types of commercialized fuel cells, but the high prices are the big barrier of this energy from becoming widespread in the market. A report on plasma treated nanostructures on GDL [111] improved the good performance of a proton exchange membrane fuel cell. Hydrophobic nanolayer was deposited on GDL surfaces via RF-PECVD with HMDSO as a precursor with different thickness. The performance of the treated GDL is much better than that of pristine GDL, due to the enhanced hydrophobicity and the increase of surface ratio.

Solar cells and photovoltaics are definitely a promising area for sustainable and green energy in the present and near future. These devices have become the strategic energy in some countries’ policies. Both use light to make electrical energy based on the photovoltaic effect. The photovoltaic is a single unit of solar cells or solar panels. The system connects to energy storage devices or electrical systems to store energy, or provide energy to loads. They both have similar mechanisms in converting the energy of light directly into electricity, which could be summarized in three steps: (1) the absorption of light, generating electron-hole pairs/excitons; (2) the separation of charged/excited carriers to positive/negative types; and (3) the separation of those charged carriers to an external circuit. Plasma treatment is used for increasing the effectiveness of all the components in each step, such as anti-reflective [112], electrode [113], and bandgap [114] for photovoltaics. Researches in solar cells are blooming with many records in efficiency. A work [115,116] on tandem architecture between organic and inorganic single-junction solar cells has achieved an efficiency of 13%, which is significant progress from the individual single junction solar cells of 7.25% and 6.2% for DSSC and CIGS, respectively. The process used Arc-plasma deposition for Pt interlayer to minimize the damage to the layers of CIGS bottom cell, based on the rough surface of the Pt layer. Togonal et al. [117] have tried to reduce the cost of heterojunction with intrinsic thin layer solar cells, by using thinner silicon wafers in compensation with an efficient light trapping scheme by several plasma deposition layer. In their work, Si nano wire arrays were fabricated by metal assisted chemical etching, and depositing a first layer of intrinsic amorphous silicon carbide in a gas mixture of SiH_4_:CH_4_ in low plasma power, a second layer of Si:H was then deposited using pure SiH_4_, and the window layer (p) a-Si:H was deposited from a gas mixture of SiH_4_/trimethylboron in argon plasma at 200 °C. After optimizing the deposition conditions, the solar cells showed good performance, with average efficiency of 12.43% and 12.9%.

## 5. Conclusions and Perspectives

This review has described plasma-based technology and its various applications in many fields, with respect to practical plasma processing and nanostructuring techniques. A variety of reports show the versatile use of plasma in solving many challenges regarding surface modification, such as increased wettability, improved adhesion for films, biofilm inactivation, and reduced prices, while reducing the limits in size of wafers in microelectronics, sterilization for tools in hospital and health care facilities, preparation of pharmaceuticals, biological treatment of stem cells, cancer, medical implants, and tissue engineering. Recently, plasma has also widened its presence in analytical methods, such as ICP-MS. The ICP-MS has paved a way to solve some difficult measurements in detecting metal or several non-metals in low concentration with good speed, precision, and sensitivity [118]. However, plasma techniques applied to nanostructuring process still have some obstacles to overcome, in order to make breakthroughs in improving performances of functional surfaces.

First, the lifetime of the functional surface or layers made by plasma is not always meeting the expectations for long-term usage. The surfaces of a bulk material always undergo changes in chemical and physical conditions, causing erosion or fouling, and resulting in destruction of its surface function. Second, the selective interactions of particles in plasma environment depend on plasma types, inert gases or complex monomers, temperature, pressure, or substrate materials, to allow the required modifications that impart properties for specific applications [119].

Both obstacles basically could be completely solved with large experience in plasma processing, deep knowledge in interactions between substrate materials with plasma gases, and in plasma particles with each other, and the effects of physical setting parameters on the system.

In general, plasma-based nanostructuring is a critical process in many applications, and still offers a large potential for vast application in the future, where the requirements will become much more complex. The vision for plasma-functional polymers, as a high technology tool, is to satisfy social demands, to focus on the strategy of finding safer and sustainable energy, water, food, and materials, and to protect human health.

## Figures and Tables

**Table 1:** Plasma nanostructuring on polymer substrate.

Applicable Field	Substrate Materials	Plasma Source/Techniques	Gas	Nanostructures	Refs.
Morphology	NR-7, SU-8 and PMMA	RIE	O_2_	Vertical nanowire structures, single level (non-hierarchical array of nanowire structures)	[30]
	PS sphere	a plasma etching	Air, Ar/O_2_	Surface roughness	[31]
	PET	RF-PECVD	O_2_	pillar- or hair-like nanostructure	[32]
	PS, PMMA	RIE	Ar/O_2_	Nanoroughening	[33]
	UV resist	Molding and plasma ashing	Ar–O_2_	moth-eye-like surface morphology	[34]
	Su-8	ICP-RIE	O_2_, SF_6_	lower roughness and higher etch rate	[35]
	PS-*r*-PDSS	RF-ICP	O_2_, H_2_/N_2_, or H_2_	Directed self-assembly nanopattern	[36]
Wettability	PDMS/PTFE	Glow discharge	CF_4_/O_2_	Nanoparticles/Superhydrophobic	[37]
	Teflon film	Glow discharge	O_2_	nanocone arrays	[38]
	PMMA	Helicon Plasma reactor	O_2_	Nanoroughness	[39]
	PDMS	Helicon Plasma reactor	SF_6_	nanotexturing	[39]
	PES	(RF) glow discharge power	CF_4_	Nanosized hollow porous structure	[40]
Bio and medical	PS	Glow discharge, RF	O_2_	micropatterned grooves and nanostructured roughness	[41]
	PLLA	IC-RF-glow discharge plasma	NH_3_	Super hydrophilic	[42]
	UHMWPE	Plasma etching-	N_2_	Rough surface	[43]
	PS	Glow discharge, RF	CF_4_/O_2_	Dense, orderly arrays nanostructures	[44]
Energy and Electronic	photoresist	ICP reactor	Cl_2_/O_2_; CF_4_/CH_2_F_2_; SO_2_/O_2_	24 nm wide gate patterns	[45]
	SU-8	Plasma etching	O_2_	Hemispherical pattern and nano-hairy structures	[46]
	HSQ resist	Plasma etching	O_2_	28 nm HSQ mask with lower width of Graphene nanowire	[47]
	PS-*b*-PDMS	RIE	CF_4_, O_2_, Ar	Surface masking nanostructures, and plasma induced doping	[48]

## Abbreviations

a-Si:H	Amorphous Silicone and Hydrogen composition
CIGS	Copper indium gallium selenide
DNA	DeoxyriboNucleic Acid
DLC	Diamond-like carbon
D	Dimension
DC	Direct current
DSSC	Dye-sensitized solar cell
F-DLC	Fluorinated DLC
FIB	Focused ion beam
GDL	Gas diffusion layer
HT	Head tail
HMDSO	Hexamethyldisiloxane
HOPG	Highly oriented pyrolytic graphite
HSQ resist	Hydrogen silsesquioxane resist
ICP	Inductively coupled plasma
ICP-MS	ICP-mass spectrometry
IB	Ion beam
MD	Molecular dynamic
NG	Natural graphite
NR-7, SU-8	Negative Resist
OSA	Oil sliding angle
PC	Polycarbonate
PECVD	Plasma enhance chemical vapor depostion
PIII&D	Plasma immersion ion implantation and deposition
PES	Polyethersulfone
PET	Polyethylene terephthalate
PLLA/PLGA	Poly- l-lactic acid/Poly(Lactide-*co*-Glycolide)
PMMA	Poly(methyl methacrylate)
PS/PI	Polystyrene/Polyimide
PS- *b*-PDMS	Polystyrene- *b*-polydimethylsiloxane
PS- *r*-PDSS	Random copolymers of polystyrene and 4-pentamethyldisilylstyrene
PVA	Polyvinil acrylate
PDMS/PTFE	Polytetrafluoroethylene/Polydimethylsiloxane
RF	Radio frequency
RgGs	Rabbit γ-Globulins
RIE	Reactive ion etching
TEM	Transmission electron microscopy
UHV	Ultra high voltage
UV resist	Ultra violet resist
UHMWPE	Ultra-high-molecular-weight polyethylene
